# Autophagy is Involved in Cardiac Remodeling in Response to Environmental Temperature Change

**DOI:** 10.3389/fphys.2022.864427

**Published:** 2022-04-19

**Authors:** C. Ruperez, A. Blasco-Roset, D. Kular, M. Cairo, G. Ferrer-Curriu, J. Villarroya, M. Zamora, F. Crispi, F. Villarroya, A. Planavila

**Affiliations:** ^1^ Departament de Bioquímica i Biologia Molecular, Institut de Biomedicina (IBUB), Universitat de Barcelona and CIBER Fisiopatología de la Obesidad y Nutrición, Barcelona, Spain; ^2^ Fetal Medicine Research Center, BCNatal -Barcelona Center for Maternal-Fetal and Neonatal Medicine (Hospital Clinic and Hospital San Juan de Deu), Institut Clinic de Ginecologia, Obstetricia i Neonatalogia, Institut d’Investigacions Biomediques August Pi i Sunyer, University of Barcelona, Barcelona, Spain

**Keywords:** autophagy, hypertrophy, heart, temperature, metabolism

## Abstract

**Objectives:** To study the reversibility of cold-induced cardiac hypertrophy and the role of autophagy in this process.

**Background:** Chronic exposure to cold is known to cause cardiac hypertrophy independent of blood pressure elevation. The reversibility of this process and the molecular mechanisms involved are unknown.

**Methods:** Studies were performed in two-month-old mice exposed to cold (4°C) for 24 h or 10 days. After exposure, the animals were returned to room temperature (21°C) for 24 h or 1 week.

**Results:** We found that chronic cold exposure significantly increased the heart weight/tibia length (HW/TL) ratio, the mean area of cardiomyocytes, and the expression of hypertrophy markers, but significantly decreased the expression of genes involved in fatty acid oxidation*.* Echocardiographic measurements confirmed hypertrophy development after chronic cold exposure*.* One week of deacclimation for cold-exposed mice fully reverted the morphological, functional, and gene expression indicators of cardiac hypertrophy. Experiments involving injection of leupeptin at 1 h before sacrifice (to block autophagic flux) indicated that cardiac autophagy was repressed under cold exposure and re-activated during the first 24 h after mice were returned to room temperature. Pharmacological blockage of autophagy for 1 week using chloroquine in mice subjected to deacclimation from cold significantly inhibited the reversion of cardiac hypertrophy.

**Conclusion:** Our data indicate that mice exposed to cold develop a marked cardiac hypertrophy that is reversed after 1 week of deacclimation. We propose that autophagy is a major mechanism underlying the heart remodeling seen in response to cold exposure and its posterior reversion after deacclimation.

## Introduction

A cold environment is known to cause hypertension and cardiac hypertrophy, leading to increases in cardiovascular mortality and morbidity. Numerous studies have demonstrated that winter is associated with higher incidence of cardiovascular disease, aggravation of hypertension, and other cardiovascular events such as infarction and stroke (Baker-Blocker, 1982; Jakovljević et al., 1996; Sheth et al., 1999). However, while the epidemiologic effects of cold weather on cardiovascular health are well stablished, little is known about the regulatory processes involved in the development of cardiac hypertrophy in response to low temperature. Cold-Induced Cardiac Hypertrophy (CICH) is the term used to describe the increment of cardiac mass due to an increase of cardiomyocyte size in response to cold exposure. This mechanism, independent of the elevation in blood pressure in the same conditions -or Cold-Induced Hypertension (CIH) (Fregly et al., 1989; Riesselmann et al., 1992)-, seems to be directly related to an up-regulation of certain factors controlling cellular growth in cardiomyocytes (Bello Roufai et al., 2007; Liang et al., 2017). On the other hand, the potential reversibility of CICH is not well known. Thus, a better understanding of the mechanisms leading to CICH and the reversion of this process might be helpful to prevent the incidence of cardiovascular mortality during winter months.

Autophagy is a self-degradative process that contributes to the degradation of cellular components; it allows cells to remove old or damaged organelles and/or provides energy substrates under nutrient deprivation (Feng et al., 2014; Li et al., 2016). In cardiomyocytes, which generally lack proliferative ability (Bergmann et al., 2009; Nakamura and Sadoshima, 2018), autophagy is especially important for maintaining homeostasis and adequate cell size and function (Bento et al., 2016). Autophagy impairment in cardiomyocytes induces cardiac hypertrophy, increasing cell size and reducing viability (Wang et al., 2012). Several studies in animal models of cardiomyopathy found alterations in the autophagic pathway (Zech et al., 2020). However, the involvement of autophagy during CICH and its reversion is not well known.

In the present study, we describe the implication of autophagy in cardiac adaptation to cold exposure and posterior deacclimation to a warmer temperature. We show that a functional autophagic pathway is necessary for the reversion of cold-induced cardiac hypertrophy upon the return to a warmer temperature.

## Methods

### Animals and Cold Exposure-Deacclimation Protocol

C57BL/6N mice were obtained from Jackson laboratory (United States). Male mice at 2–3 months of age were maintained at 4°C for 24 h (acute cold exposure, AC) or 10 days (chronic cold exposure, CC). After cold exposure, samples were obtained or animals were returned to room temperature for the deacclimation condition. Deacclimation groups were maintained at room temperature (22°C) for 24 h (acute deacclimation, AD) or 1 week (chronic deacclimation, CD) after previous exposure to 4°C for 10 days. When indicated, deacclimating mice were subjected to daily intraperitoneal (i.p.) injections with hydroxychloroquine [Chlor; 50 mg/kg body weight (b.w.)] to inhibit autophagy or PBS as a control. When indicated, leupeptin (40 mg/kg b.w.) was administered i.p. 1 h before sacrifice to block autophagy*.* The control group (CT) are animals in standard housing conditions, at 22°C. Since the difference in the age of the animals at the time of sacrifice was only ±7 days, a single control group was used for comparison with the distinct temperature environment conditions. CT animals were sacrificed at the same time than the CD group, and the weight and food intake of the animals were monitored for the duration of the experiments. Animals were housed two per cage, and b.w. and food intake were monitored during all procedures. Mice were sacrificed by cervical dislocation. Tissues were dissected and frozen for further analysis.

### Echocardiography

Animals were anesthetized with 1.5% isoflurane and cardiac parameters were assessed by echocardiography with a VividQ instrument (GE Healthcare, Piscataway, NJ, United States) equipped with a 12-MHz microprobe. Ventricular measurements in M-mode and Doppler were made the day before sacrifice. Three different cardiac cycles were measured for each assessment, and average values were obtained. Analyses of echocardiographic images were performed by two different observers in a blinded manner.

### Cardiac Histology and Quantification of Cardiomyocyte Area

After extraction, each heart was cut transversely at mid-height and the superior half was fixed in 4% formaldehyde, embedded in paraffin, and sectioned. The sections were deparaffinized and stained with hematoxylin and eosin (H&E), and posteriorly applied for the determination of cardiomyocyte size. Quantification of the areas was assessed in perpendicular cuts of the posterior left ventricular wall. Only cells in perpendicular plane were considered for the analysis. The quantification of the areas was performed with the Open Source software ImageJ, using the ROI manager tool.

Fibrosis was determined by Masson’s trichrome staining (Panreac, Barcelona, Spain). Images were obtained under an Olympus BX61 microscope.

### RNA Isolation and Real-Time Reverse Transcription Polymerase Chain Reaction

RNA extraction from tissue was performed using TriPure reagent (Roche, Indianapolis, IN, United States). Reverse transcription (RT) was performed with 0.5 μg of total RNA in a reaction volume of 20 μl, using a High Capacity RNA-to-cDNA kit (Applied Biosystems, Foster City, CA, United States). mRNA expression was determined by real-time PCR using TaqMan Gene Expression Assays (ThermoFisher Scientific, Waltham, MA, United States). The final reaction mix (25 μl) contained 1 μl cDNA, 12.5 μl TaqMan Universal PCR Master Mix (ThermoFisher Scientific), 250 nM probes, and 900 nM primers from the Assays-on-Demand Gene Expression Assay Mix or the Assays-by-Design Gene Expression Assay Mix (ThermoFisher Scientific). Each sample analysis was conducted in duplicate for increased accuracy, and the mean value was used to calculate the expression of the gene of interest and the reference gene (Cyclophilin A, *Ppia*). The mRNA levels of each gene of interest were normalized to *Ppia* using the comparative (2^−ΔCT^) method.

### Protein Level Determination by Western Blot

Whole-cell lysates or tissue homogenates were obtained in ice-cold RIPA buffer containing a protease inhibitor cocktail (Rupérez et al., 2018). Proteins were resolved by 12% SDS-PAGE and transferred to Immobilon-P membranes (Millipore). Protein detection was performed using antibodies against LC3B (Cell Signaling, Beverly, MA, United States; #2775), p62 (Cell Signaling; #5114), Atg7 (Cell Signaling; #2631) and Parkin (Cell Signaling; #2132). Ponceau staining and GAPDH (G9545, Sigma-Aldrich) were used as a loading control.

### Electron Microscopy

Cardiac samples were fixed in 2.5% glutaraldehyde and 2% paraformaldehyde in 0.1 M phosphate buffer (pH 7.4), and postfixed in 1% osmium tetroxide and 0.8% FeCNK in phosphate buffer. After dehydration in a graded acetone series, tissue samples were embedded in Spurr resin. Ultrathin sections were stained with uranyl acetate and lead citrate, and examined with a Jeol 1010 transmission electron microscope (Izasa Scientific, Barcelona, Spain).

### Statistics

Cell culture experiments were conducted in triplicate on at least three independent cardiomyocyte isolations. Groups of five mice were used for the *in vivo* experiments. Results are presented as means ± SEM. Statistical analysis was done with one-way ANOVA or two-way ANOVA followed by post-hoc tests as appropriate, using the GraphPad Prism software (GraphPad Software Inc., San Diego, CA, United States). A p-value less than 0.05 was considered statistically significant. One, two, and three symbols denote *p* < 0.05, *p* < 0.01, and *p* < 0.001, respectively.

### Study Approval

All animal procedures performed conform to the guidelines from Directive 2010/63/EU of the European Parliament on the protection of animals used for scientific purposes and approved by the Institutional Animal Care and Use Committee of the University of Barcelona.

## Results

### Chronic Cold Leads to a Cardiac Hypertrophy That is Reversed During Deacclimation

**FIGURE 1:**
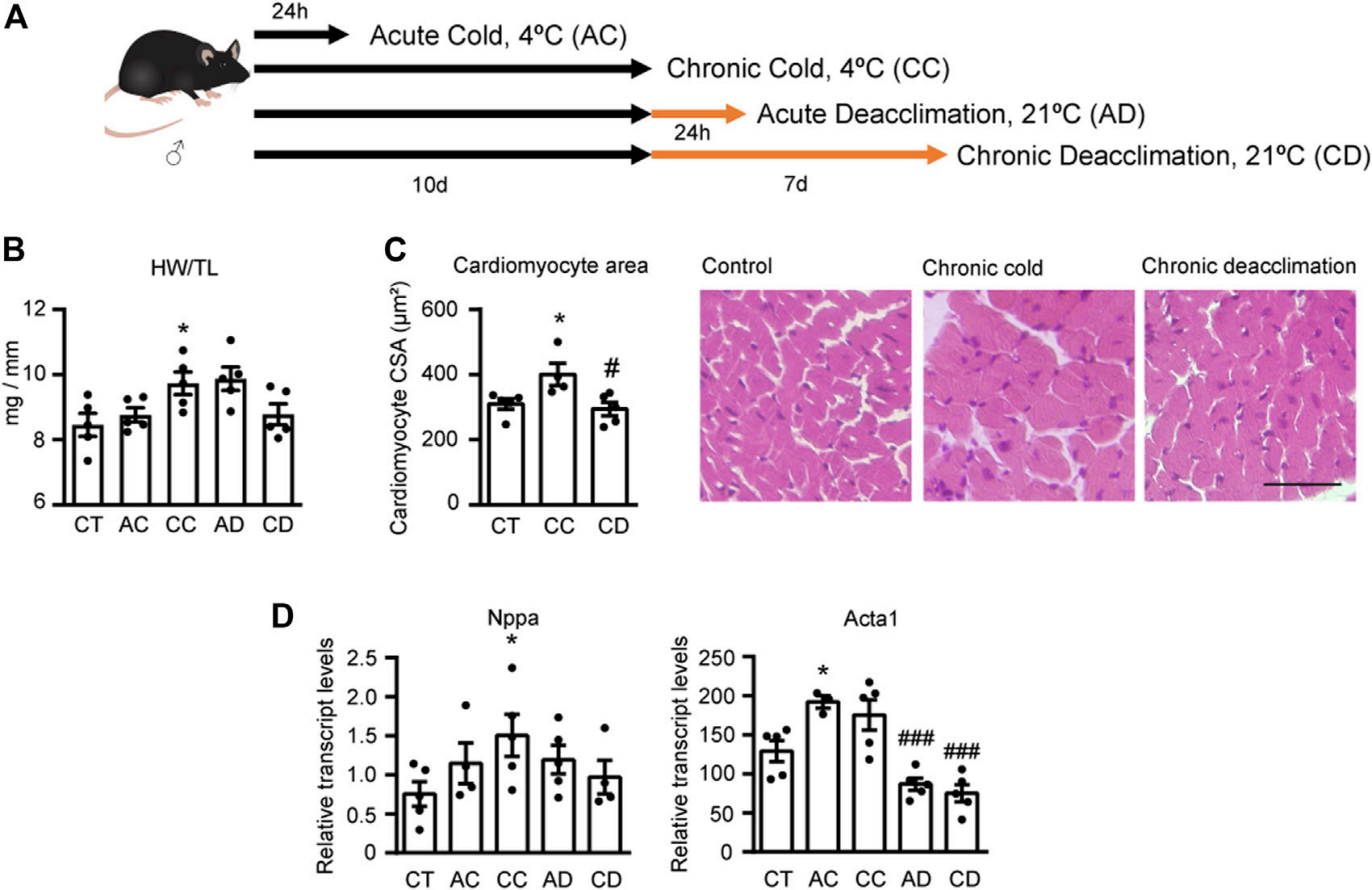
Chronic cold induces cardiac hypertrophy and deacclimation reverses this process. **(A)** Mice were subjected to acute cold (AC; 4°C, 24 h), chronic cold (CC; 4°C, 10 days) or CC followed by acute deacclimation (AD; 21°C, 24 h) or chronic deacclimation (CD; 21°C, 7 days). **(B)** Heart weight (mg) to tibia length (mm) (HW/TL) ratio in control (CT), AC, CC, AD and CD mice. **(C)** Quantification of cardiomyocyte area in the left ventricular wall (*left*) and representative histological sections of H&E-stained hearts, which were used for the determination of cardiomyocyte area (*right*). Magnification, 20×. Scale bar: 50 μm. **(D)** mRNA expression levels of the hypertrophy markers, *Nppa* and *Acta1* normalized to *Ppia*. Data were analyzed by one-way ANOVA. Results are presented as means ± SEM (*n* = 5 mice/group; *p < 0.05 compared with control mice, and ^#^ *p* < 0.05 compared with CC mice). One, two, and three symbols denote *p* < 0.05, *p* < 0.01, and *p* < 0.001, respectively.

Three-month-old mice maintained at 21°C were subjected to acute cold (AC; 4°C, 24 h), chronic cold (CC; 4°C, 10 days), or 10 days of cold exposure followed by acute deacclimation (AD; 21°C, 24 h) or chronic deacclimation (CD; 21°C, 7 days) at room temperature. Thereafter, hearts were analyzed (Figure 1A). We found that chronic cold exposure significantly increased the heart weight/tibia length (HW/TL) ratio (a measure of cardiac hypertrophy), whereas mice subjected to cold followed by chronic deacclimation showed a HW/TL ratio similar to that of non-cold-challenged mice (Figure 1B), indicating reversion. No changes in HW/TL ratio were observed in acute conditions (AC or AD). Histological examination of hematoxylin and eosin (H&E)-stained left ventricle tissue sections revealed that the cardiomyocyte cross-sectional area (CSA) was significantly increased in mice after cold exposure, indicating cellular cardiac hypertrophy (Figure 1C). In contrast, the CSA of mice subjected to cold exposure followed by deacclimation was significantly decreased from the post-cold-exposure level to that seen in non-cold-exposed mice.

Next, we assessed the mRNA expression levels of the hypertrophy marker genes encoding atrial natriuretic factor (*Nppa*) and α-actinin (*Acta1*) in the hearts of mice subjected to AC, CC, AD, and CD (Figure 1D). We found that CC mice exhibited significantly increased *Nppa* expression levels, and deacclimation caused *Nppa* expression to progressively normalize to levels similar to those seen in non-cold-exposed mice. Both AC and CC increased the transcript levels of *Acta1*, although the increase was statistically significant only for AC. Deacclimation significantly decreased these upregulations of *Acta1* mRNA expression.

Finally, we performed echocardiographic measurements to determine cardiac function in these animals (Table 1). We found that the interventricular septum (IVS) and left ventricular internal diameter (LVID) in both systole and diastole were significantly increased in the CC group, indicating the development of cardiac hypertrophy. No difference was found in the left ventricular posterior wall dimensions (LVPW). During deacclimation, IVSd and LVPWd in diastole, and LVPWs in systole were significantly reduced compared with those seen in cold-exposed mice, indicating reversion of the cold-induced hypertrophic process. Finally, the measures of cardiac function, LV ejection fraction (EF) and fractional shortening (FS), were significantly decreased by cold exposure and significantly recovered after deacclimation.

**TABLE 1:** Echocardiographic data from mice.

	Control (CT)	Chronic cold (CC)	Chronic deacclimation (CD)	Chronic deacclimation (CD) + chlor
IVSd (mm)	0.58 ± 0.01	0.72 ± 0.01^****^	0.62 ± 0.01^$$$$^	0.68 ± 0.01^****,#^
LVPWd (mm)	0.69 ± 0.03	0.72 ± 0.01	0.65 ± 0.02^$$^	0.71 ± 0.02
LVIDd (mm)	4.21 ± 0.08	4.59 ± 0.08^*^	4.66 ± 0.08^*^	4.24 ± 0.01^$,#^
IVSs (mm)	0.92 ± 0.03	1.05 ± 0.02^*^	1.05 ± 0.05	0.98 ± 0.03
LVPWs (mm)	0.93 ± 0.03	0.92 ± 0.01	0.82 ± 0.03^*,$^	0.92 ± 0.03
LVIDs (mm)	3.08 ± 0.07	3.54 ± 0.07^**^	3.46 ± 0.14^*^	3.29 ± 0.05
EF (%)	59.33 ± 1.12	50.00 ± 1.12^***^	57.25 ± 2.72^$^	51.75 ± 1.55^*^
FS (%)	27.17 ± 0.65	22.10 ± 0.74^**^	26.00 ± 1.78^$^	22.25 ± 0.63^*^

All measurements are expressed as mean ± SEM; *n* = 5 mice/group. Data were analyzed by one-way ANOVA; *p < 0.05 vs. control mice, #*p* < 0.05 vs. mice under deacclimation, and $*p* < 0.05 vs. mice under cold. One, two, and three symbols denote *p* < 0.05, *p* < 0.01, and *p* < 0.001, respectively. Interventricular septal dimension, Left ventricle posterior wall, and left ventricular internal diameter after diastole are abbreviated IVSd, LVPWd, and LVIDd, respectively, and those after systole are abbreviated IVSs, LVPWs, and LVIDs, respectively. Other abbreviations are ejection fraction, EF (%), fractional shortening; FS (%); Chlor, hydroxychloroquine.

Based on these findings, we concluded that chronic cold exposure for 10 days leads to development of cardiac hypertrophy, and that this process is reversed when mice are returned to room temperature.

### Cardiac Metabolism and Fibrosis are Altered Under Cold and Deacclimation

**FIGURE 2:**
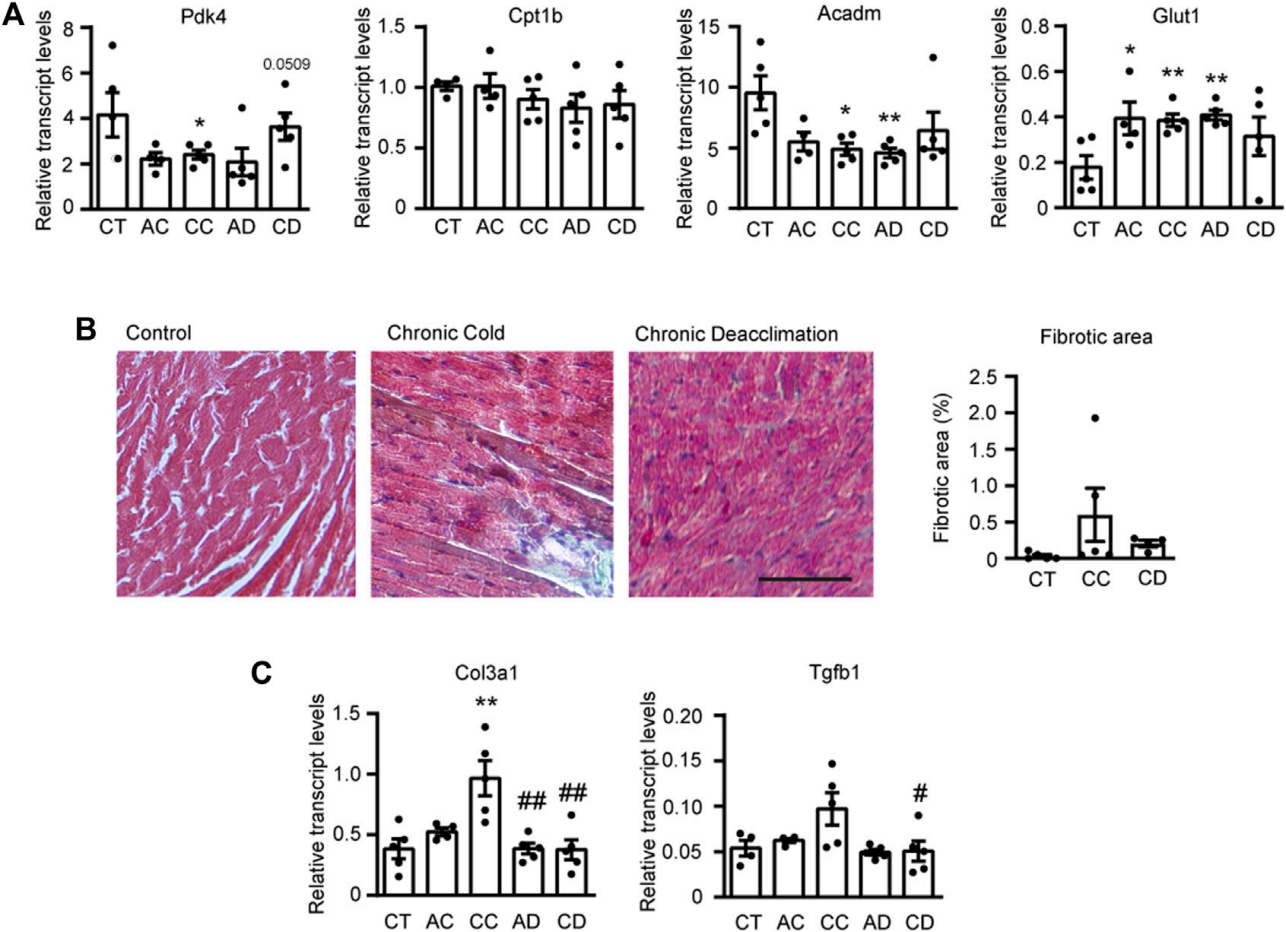
Cardiac metabolism and fibrosis are altered under cold and deacclimation. Mice were subjected to acute cold (AC; 4°C, 24 h), chronic cold (CC; 4°C, 10 days) or CC followed by acute deacclimation (AD; 21°C, 24 h) or chronic deacclimation (CD; 21°C, 7 days). **(A)** mRNA expression levels of the fatty acid oxidation-related genes, *Pdk4*, *Cpt1b*, and *Acadm*, and the glucose transporter gene, *Glut1* normalized to *Ppia*. **(B)** Representative images showing Masson’s trichrome staining (*left*) and quantification of left ventricular fibrotic areas, calculated as the positively stained areas divided by the total area of the heart section (*right*). Scale bar: 100 μM. **(C)** mRNA expression levels of the fibrotic markers, *Col3a1* and *Tgfb1* normalized to *Ppia.* Data were analyzed by one-way ANOVA. Results are presented as means ± SEM (*n* = 5 mice/group; *p < 0.05 compared with control mice, and ^#^ *p* < 0.05 compared with CC mice). One, two, and three symbols denote *p* < 0.05, *p* < 0.01, and *p* < 0.001, respectively.

Given that there is a close association between impaired fatty acid oxidation (FAO) and cardiac hypertrophy (Planavila et al., 2011; Rupérez et al., 2021), we next examined the expression levels of FAO-related genes and that encoding the glucose transporter, Glut1 (Figure 2A). We found that the transcript levels of the FAO-related genes encoding pyruvate dehydrogenase kinase-4 (*Pdk4*) and medium-chain acyl-CoA dehydrogenase (*Acadm*) were significantly decreased in mice subjected to chronic cold and reversed after chronic deacclimation back to room temperature. We did not find any significant difference for the FAO-related gene encoding carnitine palmitoyltransferase (*Cpt1b*). Conversely, *Glut1* was significantly increased in mice after prolonged cold exposure, and this alteration was reverted after deacclimation. These data indicate that during chronic cold, the cardiac FAO machinery is reduced but glucose uptake is increased in the myocardium, and these changes are partly reversed after deacclimation from a cold environment to a warmer one.

Next, we assessed cardiac fibrosis by performing Masson’s trichrome staining and evaluating the transcript expression levels of fibrosis-related genes encoding collagen 3 (*Col3a1*) and transforming growth factor-β (*Tgfb1*) (Figures 2B,C). Quantification of fibrotic areas showed that there was less fibrosis during deacclimation than under chronic cold, although the difference was not statistically significant (Figure 2B). However, the expression levels of fibrotic genes *Col3a1* and *Tgfb1* were increased after chronic exposure to cold and significantly reduced back to pre-cold levels after deacclimation (Figure 2C).

### Cardiac Autophagy is Blocked Under the Chronic Cold Condition and Reactivated After Acute Deacclimation

**FIGURE 3:**
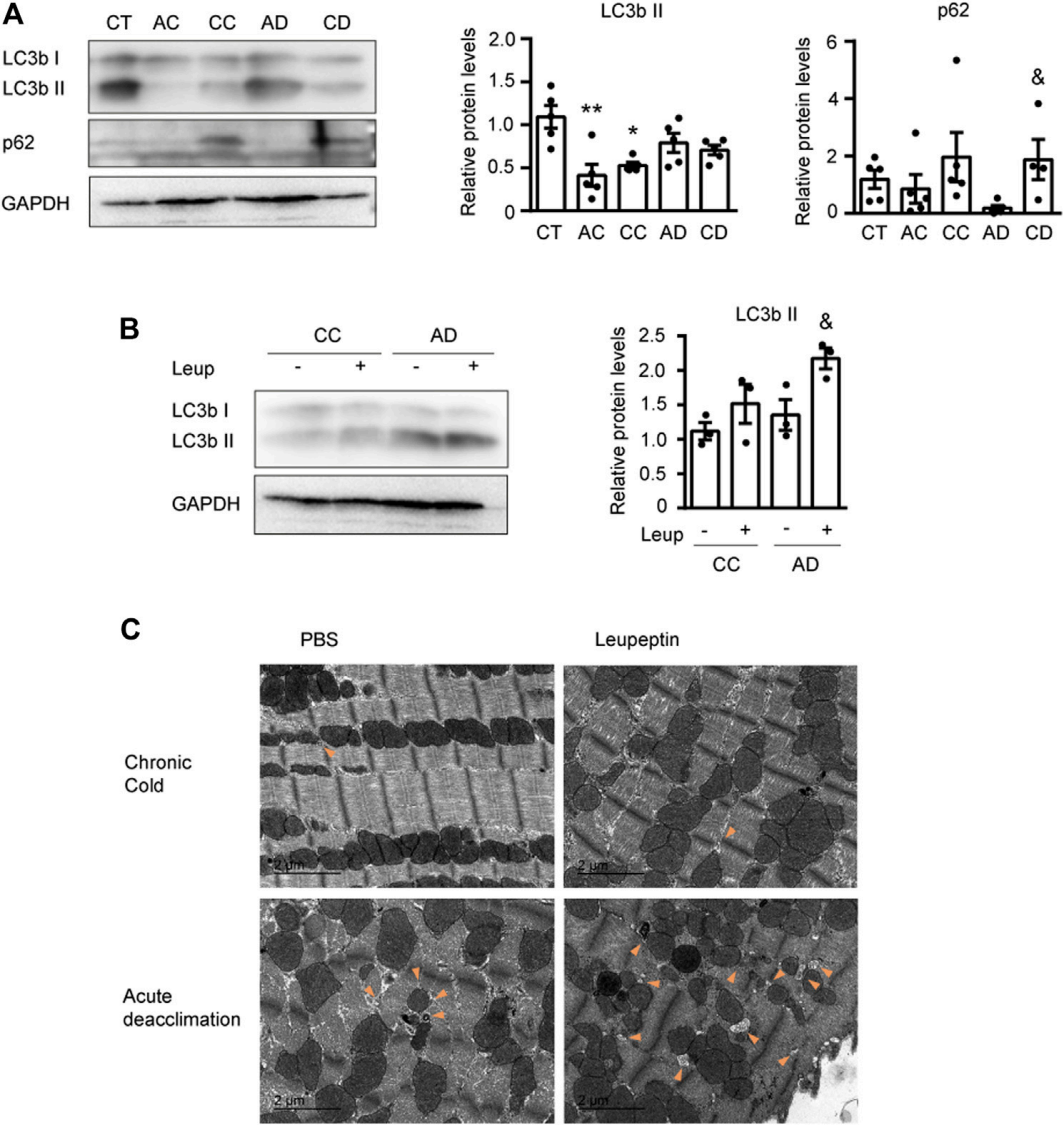
Cardiac autophagy is modulated by cold and deacclimation. Mice were subjected to acute cold (AC; 4°C, 24 h), chronic cold (CC; 4°C, 10 days) or CC followed by acute deacclimation (AD; 21°C, 24 h) or chronic deacclimation (CD; 21°C, 7 days). **(A)** Representative image (*left*) and quantification (*right*) of the immuno-blot analysis of LC3b and p62 protein levels in the heart. GAPDH was used as loading control (*n* = 5 mice/group). **(B)** Western blot analysis of LC3b protein levels in mice subjected to CC or AD with or without the application of leupeptin (Leup) 1 h before sacrifice. GAPDH was used as loading control (western blot was performed using samples from three different mice per group). **(C)** Representative transmission electron microscopy images of hearts from mice subjected to CC or AD and treated with or without leupeptin. Orange arrowheads indicate autophagosomes. Scale bar: 2 μm. (*n* = 5 mice/group). Results are expressed as means ± SEMs. Data were analyzed by one-way ANOVA **(A)** and two-way ANOVA **(B)** (*p < 0.05 compared with control [CT] mice; ^&^ *p* < 0.05 compared with AD mice). One, two, and three symbols denote *p* < 0.05, *p* < 0.01, and *p* < 0.001, respectively.

To determine the molecular mechanism responsible for the remarkable cardiac plasticity seen in response to variation in environmental temperature, we next analyzed the autophagic process. Autophagy, which is a molecular mechanism responsible for the degradation of intracellular components, has been related to cardiac hypertrophy (Kaludercic et al., 2020). First, to determine autophagic activity, we examined the protein levels of phosphatidylethanolamine-conjugated microtubule-associated protein one light chain three beta (LC3bI and LC3bII) and the substrate of autophagic degradation, p62 (Figure 3A). Immunoblotting analyses showed that cold exposure (AC and CC) significantly reduced the level of LC3bII and CC increased the level of p62, suggesting that autophagy is reduced under cold exposure. During deacclimation (AD and CD), the level of LC3bII was unchanged, but that of p62 was dramatically reduced after AD and fully recovered after CD. Collectively, these data suggest that sustained cold exposure blocks autophagy and deacclimation appears to reactivate this process fairly quickly.

To confirm these findings, we treated mice with the autophagy inhibitor, leupeptin (Leup) just before sacrifice, and analyzed the autophagic flux (Figure 3B). We found that the LC3bII protein level was not altered by leupeptin treatment of cold-exposed mice, indicating that cold exposure did not trigger autophagic activation. However, LC3bII was accumulated in leupeptin-treated mice subjected to acute deacclimation, compared to leupeptin-untreated mice. This indicates that the autophagic flux was activated after 24 h of deacclimation from cold back to room temperature.

Finally, we used transmission electron microscopy to examine the subcellular structures of heart sections from mice subjected to cold and deacclimation, with or without leupeptin treatment (Figure 3C). In mice subjected to chronic cold, we observed very few degradation vesicles whose structures were consistent with that of an autophagosome. In contrast, we observed numerous autophagosome-like degradation vesicles in mice subjected to acute deacclimation, especially in those treated with leupeptin. This is consistent with activation of the autophagic flux.

Collectively, these results indicate that autophagy is blocked after prolonged cold exposure, but reactivated at 24 h after the mice are returned to room temperature.

### Autophagy Participates in the Hypertrophy Reversion Associated With Cold Deacclimation

**FIGURE 4:**
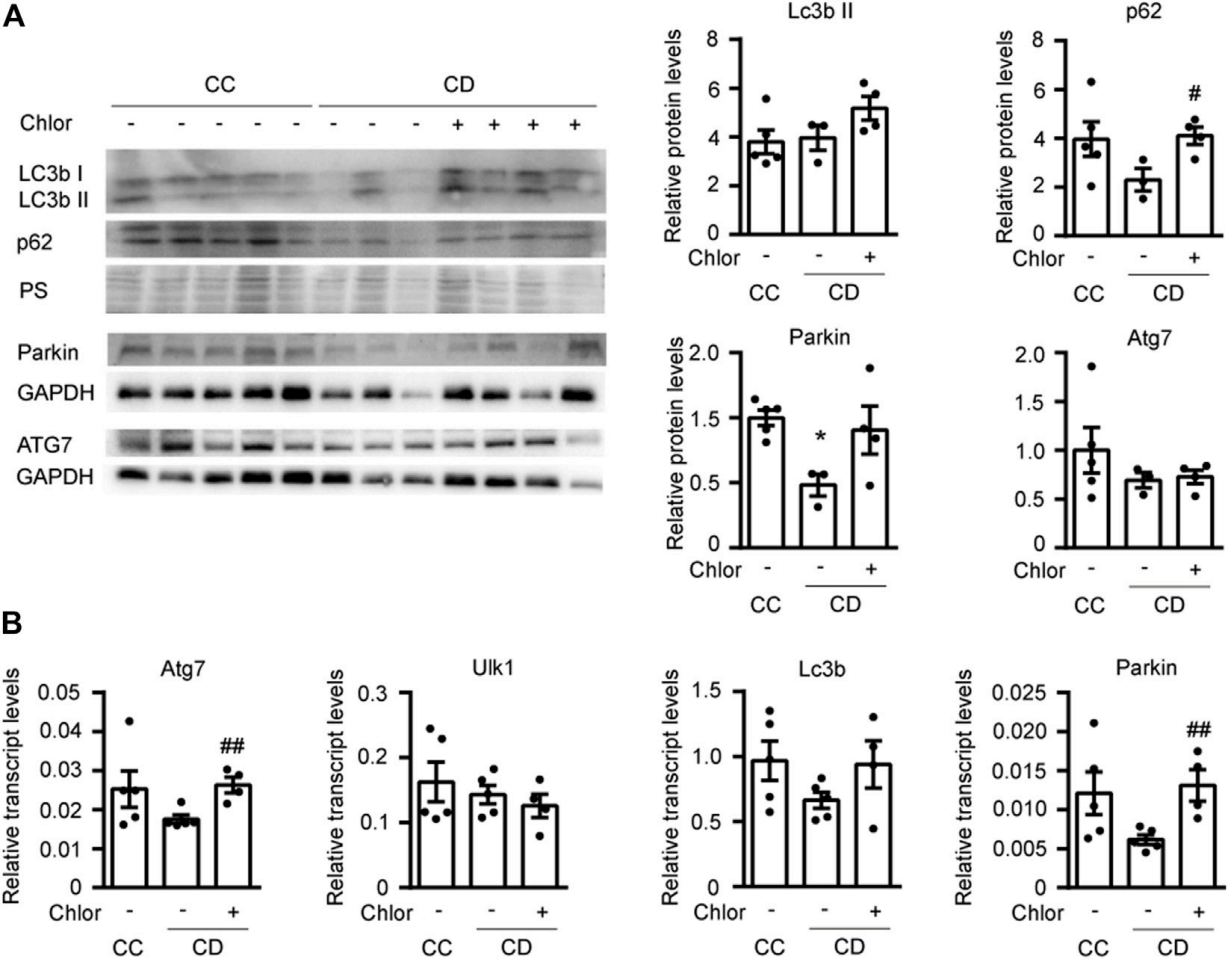
Cardiac autophagy is blocked by 1 week of hydroxychloroquine (Chlor) treatment during deacclimation. Mice were exposed to cold (CC; 4°C, 10 days) or cold followed by deacclimation (CD; 21°C, 7 days). During deacclimation, mice of the CD group were subjected to daily intraperitoneal (i.p.) injection of vehicle (PBS) or hydroxychloroquine (Chlor) for 1 week. **(A)** Representative image (*left*) and quantification (*right*) of the immunoblot analysis of LC3b, p62, Atg7 and Parkin protein levels in the heart. Ponceau staining (PS) and GAPDH were used as loading control (western-blot was performed using heart samples from five mice in CC, three mice in AD and four mice in AD + Chlor). **(B)** Expression levels of the autophagy-related genes, *Atg7*, *Ulk1*, *Lc3b*, and *Parkin*, normalized to *Ppia* in the myocardium (*n* = 5 mice/group). Results are expressed as means ± SEMs. Data were analyzed by one-way ANOVA (^#^ *p* < 0.05 compared with PBS-treated deacclimated mice). One, two, and three symbols denote *p* < 0.05, *p* < 0.01 and *p* < 0.001, respectively.

In order to determine whether activation of autophagy during deacclimation from cold is responsible for the reversion of the hypertrophic process, we subjected 2-month-old mice to a daily intraperitoneal (i.p.) injection with the autophagy inhibitor hydroxychloroquine (Chlor) for 1-week in order to fully inhibit autophagy at the time of deacclimation (Figure 4). To confirm the effect of Chlor, we first analyzed the protein levels of LC3b and p62 (Figure 4A). In animals subjected to deacclimation and treated with Chlor, the levels of both LC3bII and p62 were higher (statistically significant in the case of p62) than in deacclimated mice treated with PBS, indicating that the autophagic flux was effectively inhibited by Chlor. The protein levels of Parkin were significantly reduced in deacclimated mice treated with PBS and the protein levels of Atg7 were unchanged. To further confirm inhibition of the autophagic process, we also analyzed the expression levels of genes involved in this process, such as *Atg7, Ulk1, Lc3b*, and *Parkin* (Figure 4B). We found that the mRNA levels of *Atg7, Lc3b*, and *Parkin* tended to decrease during deacclimation from cold, compared to those seen in mice under cold exposure. In deacclimated mice treated with Chlor, in contrast, the expression levels of *Atg7, Lc3b*, and *Parkin* were increased compared to those in Chlor-untreated deacclimated mice (statistically significant for *Atg7* and *Parkin*). No change was observed for *Ulk1*. The increased levels of autophagic genes in Chlor-treated mice indicates a compensatory mechanism of the cell in order to respond to the autophagy blockade. These data further confirm that Chlor treatment successfully blocked autophagy in our system.

**FIGURE 5:**
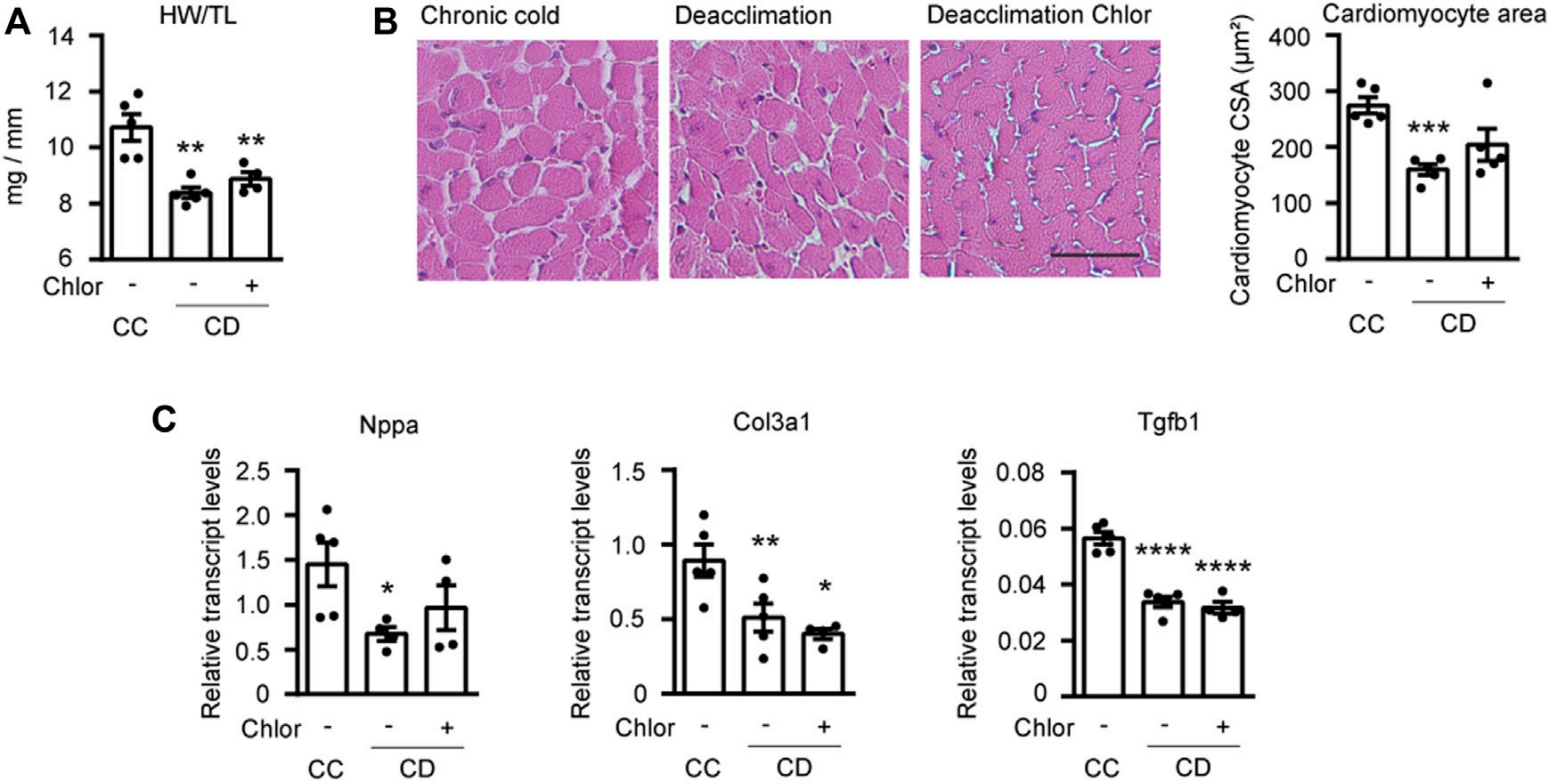
Reversion of hypertrophy is impaired by blockade of autophagy during deacclimation. Mice were exposed to cold (CC; 4°C, 10 days) or to cold followed by deacclimation (CD; 21°C, 7 days). During deacclimation (CD) animals were subjected to a daily intraperitoneal (i.p.) injection with vehicle (PBS) or hydroxychloroquine (Chlor) for 1-week. **(A)** Heart weight (mg) to tibia length (mm) ratio in CC and CD mice treated or not with Chlor. **(B)** Quantification of cardiomyocyte area in the left ventricular wall (*left*) and representative histological sections of hearts stained with H&E, which were used to determine cardiomyocyte area (*right*). Magnification, 20×. Scale bar: 50 μm. **(C)** mRNA expression levels of the hypertrophy marker *Nppa* and the fibrotic markers *Col3a1* and *Tgfb1* normalized to *Ppia.* Data were analyzed by one-way ANOVA. Results are presented as means ± SEM (*n* = 5 mice/group; *p < 0.05 compared with CC mice, and ^#^ *p* < 0.05 compared with PBS-treated CD mice). One, two, and three symbols denote *p* < 0.05, *p* < 0.01, and *p* < 0.001, respectively.

Next, we analyzed hypertrophy in the hearts of mice treated as described above (Figure 5). We found that the HW/TL ratio tended to be higher in deacclimated mice treated with Chlor than in non-Chlor-treated deacclimated mice, although this difference was not statistically significant (Figure 5A). When we analyzed the area of cardiomyocytes in these hearts, we found that mice subjected to deacclimation after previous cold exposure had significantly larger cardiomyocyte areas following Chlor treatment, compared to those in non-Chlor-treated mice (Figure 5B). We also assessed the transcript levels for markers of hypertrophy and fibrosis (Figure 5C). As expected, the mRNA level of the hypertrophy marker, *Nppa,* was significantly reduced in non-Chlor-treated deacclimated mice but not in Chlor-treated deacclimated mice, as compared to mice under cold exposure. No change was observed in the fibrotic markers, *Col3a1* and *Tgfb1*, between Chlor-treated versus non-Chlor-treated CD mice*.* Echocardiographic measurements showed that IVSd, LVPWd, LVPWs, EF, and FS were significantly reduced during deacclimation from cold in non-Chlor-treated deacclimated mice but not in Chlor-treated deacclimated mice, compared to cold-exposed mice (Table 1). Collectively, these data indicate that autophagy is involved in the deacclimation-induced reversion of the cold-induced hypertrophic phenotype.

## Discussion

Epidemiological studies have highlighted the importance of environmental temperature for cardiac health, but the effects of ambient temperature on cardiac plasticity and the underlying molecular mechanisms are not well understood. Here, we report that cold exposure leads to cardiac hypertrophy, and a 1-week deacclimation completely reverses this process. Moreover, we identify autophagy as a key process involved in the cardiac plasticity seen under alteration of environmental temperature: it is blocked during cold exposure and reactivated after deacclimation in our system.

Exposure to low temperature represents a great challenge for human health, with myocardial injury being one of the most relevant problems. The cardiac alterations associated with cold stress have been widely studied in animal models, mainly in rats (Bello Roufai et al., 2007; Cheng and Hauton, 2008; Liang et al., 2017; Kong et al., 2020), but the underlying molecular mechanism(s) and/or reversibility of these alterations have not yet been determined. In the present study, we show that 10 days of cold exposure in mice is associated with increases in the heart weight, cardiomyocyte area, and left ventricular mass, which is consistent with a hypertrophic cardiac phenotype. Moreover, our evaluation of cardiac function shows that cold stress decreases the ejection fraction and fractional shortening of mouse hearts, dampening left ventricular function. This was consistent with previously reports in other animal models subjected to cold (Cheng and Hauton, 2008; Zhang et al., 2012; Jiang et al., 2013). Whereas cold exposure-associated hypertrophy is relatively well known, its potential reversibility has received less research attention. We herein demonstrate that a 1-week deacclimation of cold-exposed mice back to room temperature could completely reverse the developed cardiac hypertrophy and associated cardiac dysfunctions. This highlights the remarkable plasticity that enables the heart to adapt to changes in environmental temperature. To our knowledge, this is the first report to examine this phenomenon in mice. Our data indicate that autophagy is activated quickly in this process: It is first seen at 24 h of deacclimation and declines 1 week later. Moreover, we show that the reversion of several hypertrophy-associated parameters are attenuated in mice in which autophagy has been blocked, suggesting that the temperature-related reversibility of hypertrophy is autophagy-dependent.

Our data agree with previous studies suggesting that autophagy is blocked during hemodynamic stress-induced remodeling of the heart, and its activation might exert protective effects (Nakai et al., 2007; Zhu et al., 2007). These findings contrast with reports suggesting that autophagy is activated during cold induced hypertrophy (Jiang et al., 2013; Kong et al., 2020; Wang et al., 2020). This discrepancy may be related to differences in the extent and duration of the studied cold stress. Based on our present data, we propose that the reactivation of autophagy is a major process contributing to the reversion of cardiac damage.

Under chronic cold, brown adipose tissue increases its demand for fatty acids to sustain non-shivering thermogenesis, causing the main metabolic substrates that are supplied to the heart to shift from fatty acids to glucose (Hauton et al., 2009; Templeman et al., 2010). The downregulation of genes involved in FAO and the increased uptake of glucose observed under chronic cold in the present work is in accordance with this scenario. Our further finding that a 1-week deacclimation partly reverses the expression levels of FAO-related genes in accordance with reversion of the hypertrophic phenotype indicates that the cardiac plasticity seen in response to environmental temperature change involves adaptive flexibility in metabolic substrate usage.

Previous studies revealed that there is overt fibrosis in the myocardium of cold stress-exposed mice (Nagasawa et al., 2016; Liang et al., 2017; Cong et al., 2018). Consistent with this, we observed that cold-exposed mice exhibited increased levels of fibrotic genes (e.g., *Tgfβ* and *Col3a1*) and a trend toward overt fibrosis. During deacclimation of cold-exposed mice, we observed reversion of this fibrosis in the myocardium, suggesting that under these conditions cardiac plasticity involves not only cardiac hypertrophy but also reversion of cold-induced cardiac fibrosis.

Collectively, our results establish the reversibility of cold-induced cardiac hypertrophy and identify autophagy as a key process involved in this adaptive cardiac plasticity process. Thus, cardiac autophagy may be viewed as a potential target in efforts to prevent the higher incidence of cardiovascular mortality during winter months. Several therapeutic strategies have been proposed to stimulate autophagy as a means to improve various diseases conditions [see Kaludercic et al. (2020) for a recent review(18)], but most of the autophagy-inducing drugs developed to date also impact other molecular pathways, which limits their therapeutic use. Our present results highlight the importance of pursuing research on the therapeutic modulation of autophagy in the context of promoting cardiac hypertrophy reversion and its subsequent benefits in the treatment of cardiac diseases.

## Data Availability

The original contributions presented in the study are included in the article/Supplementary Materials, further inquiries can be directed to the corresponding author.
